# Invasion Dynamics of Teratogenic Infections in Light of Rubella Control: Implications for Zika Virus

**DOI:** 10.1371/currents.outbreaks.873427b89ab9c75eb90c8ddb8d8c7c90

**Published:** 2016-08-16

**Authors:** C. Jessica E. Metcalf, Alan Barrett

**Affiliations:** Department of Ecology and Evolutionary Biology, Princeton University, Princeton, New Jersey, USA; Department of Pathology and Sealy Center for Vaccine Development, University of Texas Medical Branch, Galveston, Texas, USA

## Abstract

Introduction: The greatest burden for a subset of pathogens is associated with infection during pregnancy. Evidence for teratogenic effects of Zika Virus have highlighted the importance of understanding the epidemiology of such pathogens. Rubella is perhaps the most classic example, and there is much to be learned from the long history of modelling associated with this virus.

Methods: We extended an existing framework for modeling age-specific dynamics of rubella to illustrate how the body of knowledge of rubella dynamics informs the dynamics of teratogenic infections more broadly, and particularly the impact of control on such infections in different transmission settings.

Results: During invasion, the burden in women of childbearing age is expected to peak, but then fall to low levels before eventually levelling out. Importantly, as illustrated by rubella dynamics, there is potential for a paradoxical effect, where inadequate control efforts can increase the burden.

Conclusions: Drawing on the existing body of work on rubella dynamics highlights key knowledge gaps for understanding the risks associated with Zika Virus. The magnitude and impacts of sterilizing immunity, plus antigenic maps measuring cross-protection with other flaviviruses, and the magnitude of transmission, as well as likely impact of control efforts on transmission are likely to be key variables for robust inference into the outcome of management efforts for Zika Virus.

## Introduction

The impacts of rubella infection during pregnancy have been recognized since the pioneering work of Norman Gregg following introductions of this pathogen into communities in Australia during the Second World War^1^. Theoretical research into the characteristics of the population burden of pathogens with this unique manifestation followed^2^; and the teratogenic outcomes of rubella^3^ and their implications across diverse demographic, epidemiological and control settings^4^^,^ 5 ^,^ 6 ^,^ 7 are currently well described. In particular, in an endemic setting, incomplete vaccination coverage can lead to an increase in the teratogenic burden associated with rubella infection (Congenital Rubella Syndrome, or CRS), an outcome also known as the ‘paradoxical effect’. This occurs because infection with rubella provides lifelong immunity, so that in the absence of vaccination, the average age of infection can be low. Vaccination tends to increase the average age of infection by making the infection rare; and if coverage has not sufficiently reduced incidence to offset this increase, the outcome can be an increase in the burden of CRS^2^^,^ 7. Perhaps the greatest impact theoretical modeling has ever had on public health^8^ was the resulting restriction of introduction of the rubella-containing vaccine into many parts of the world^9^.

Other than some suggestive patterns from the veterinary literature with Japanese encephalitis and Wesselbron viruses 10, the flaviviruses were not known to be teratogenic; however, there is now compelling evidence that Zika virus (ZIKV) is teratogenic 11 ^,^ 12. Therefore, the existing body of work on rubella may provide powerful insights into core directions for global control efforts for management of ZIKV. Leveraging the existing knowledge of rubella dynamics to understand ZIKV burden and risk requires understanding similarities and differences between these two pathogens. In particular, how does the epidemiology of rubella map onto what is known of the dynamics of ZIKV?

Two broad scenarios are possible for ZIKV: i) **epidemic** transmission between humans via the bite of a mosquito (as for dengue virus); or ii) a predominantly **zoonotic** cycle involving an animal reservoir and a mosquito host, where humans are incidental dead-end hosts (as for West Nile virus [WNV]). At the present time, it is unknown whether human-mosquito-human transmission can be sustained, or whether a sylvatic transmission cycle will contribute to outbreaks in the Americas. A sylvatic cycle has received relatively less attention in the Americas but has been considered^13^ . Low levels of viremia detected in humans, which might indicate low probability of onward transmission (further detailed below); combined with historical suggestions that ZIKV persistence across a variety of settings might require hosts beyond the primate (human and monkey) hosts characterized 14; and highly variable prevalence across sero-surveys in human populations across Africa^15^ suggestive of erratic extinction-reintroduction dynamics all suggest that this possibility should not be discounted before more data becomes available. Sexual, blood transfusion, or similar transmission routes have also been reported, but their relevance for similar pathogens (e.g., WNV) suggests a relatively minor contribution to dynamics, although this may change as we obtain more information. Natural wild-type ZIKV infection is also likely to induce life-long homotypic immunity, as observed for infections by other flaviviruses such as yellow fever^22^ , which is also the case for infection with rubella virus^2^; nonetheless, further research into this aspect of the biology via longitudinal serological surveys are an important direction for future research (see below).

Here, we develop a simple model to illustrate how current theory on rubella may inform risk and outcomes of control for ZIKV, based on two simple transmission scenarios (epidemic and zoonotic), and interpret what this analysis suggests about core areas for further research, specifically for ZIKV, and in the context of teratogenic infections more broadly.

## Methods and Results

**Figure 1:**
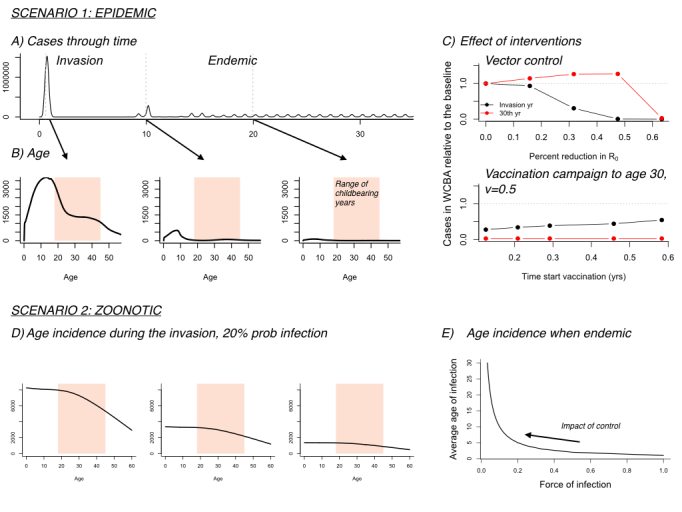
Figure 1: Epidemic scenario A) Simulation of an immunising infection (R0 = 3), from invasion to endemicity (±15th year); B) Corresponding age incidence through time (arrows) indicating reduced case burden during childbearing ages (CBA, light red), as population immunity establishes; C) Effect of interventions measured by the ratio of cases occurring during potential CBA (y axis), comparing reductions in R0 (x axis), reflecting of vector control (upper plot: a positive effect in the invasion year, but the classical paradoxical effect in endemic years, as for rubella); and the effect of a single vaccination campaign occurring in the first outbreak year, but at increasing delays (x axis), targeting ages up to 30, and with low coverage (lower plot: a consistently beneficial effect, especially for interventions in the first year; since our focus is 30 years in the future, many women will have been protected by that single campaign, despite low coverage). Zoonotic scenario D) Age incidence assuming that following pathogen emergence, 20% of the susceptible population is at risk of being infected in each time-step, shown across time-steps 1, 5, and 9; and E) Expected average age of infection (y axis) in the endemic state as a function of the rate at which susceptible individuals become infected (x axis). Control efforts such as vector control are liable to reduce the force of infection, and can thus increase the average age of infection in the endemic state.

Assuming sterilizing immunity following wild-type infection, for the **Epidemic** scenario, a simple human-to-human transmission process can approximate the dynamics of vector-transmitted infections^16^, allowing us to borrow directly from the theory relating to rubella (age structured model described in detail in^7^; here deployed with monthly age classes up to age 15, and annual thereafter). Much of this theory hinges on the value of R_0_, or the number of new infections per infectious individuals in a completely immunizing population; R_0_>1 in our Epidemic scenario, which shapes both the average age of infection, and the impact of control strategies 7. Figure 1A,B indicates basic expectations for an immunizing pathogen in endemic and invasive contexts with an R_0_ around 3 (based on analyses of the Yap outbreaks^17^ ; and in Colombia^18^ ; Figure 1A, 1B). Where the infection is endemic, the average age of infection is low, since much of the older population is protected by immunity; where the infection is invasive, all age ranges are affected, as observed during the invasion dynamics of ZIKV such as occurred in Yap^19^.

For the **Zoonotic** scenario, where humans are likely dead end hosts, R_0_<1 or even zero for human-vector-human transmission. Consequently, the key driver of the burden of congenital disease is fluctuations in the force of infection on humans resulting from transmission in the zoonotic reservoir. During pathogen invasion, every human individual is susceptible, and high burdens might occur, as observed in Yap 19 . Conversely, where exposure has consistently been high, the average age of infection might be low, and the burden correspondingly low (Figure 1E), although this will depend on the magnitude of transmission into the human population – low transmission to human susceptibles can be associated with high average age. If local extinction and reintroduction are the rule (as for many vector-borne infections) populations may remain vulnerable, with high proportions of the older population being susceptible. As in the epidemic scenario, vector control may affect the average age of infection, and should be evaluated carefully (Figure 1E). The power of vaccination, again, is the opportunity of providing direct protection to those most vulnerable.

## Discussion

Our analysis indicates similarities with core predictions that emerge from the existing body of work on rubella across the two major scenarios for ZIKV transmission. Is ZIKV more like dengue (scenario 1) or WNV (scenario 2)? The rarity of human ZIKV isolates compared to the number of infected individuals (Lanciotti et al.^20^ failed to make any isolates during the Yap outbreak) suggests a low and/or transient viremia, making onward transmission seem unlikely, suggestive more of a similarity with WNV. This accords with historic evidence of the importance of zoonotic reservoirs, e.g., in many parts of West Africa 15. Conversely, models fitted to more recent outbreaks^17^ suggest greater similarity with dengue. Overall, there is still much to be done to understand the transmission cycle of ZIKV in the Americas.

In both scenarios, however, a rapidly changing burden over the course of the early phase of the invasion, followed by endemic establishment of relatively lower burden of disease is suggested by simple modeling, and analogy with rubella. Quantifying both magnitude and timing of this shifting burden will require a deeper understanding of the underlying magnitude of transmission as well as the size of the susceptible population which is at risk, likely to be a function of both the landscape of immunity, and vector dynamics^11^. Our model illustrates that for ZIKV, as for rubella, the potential paradoxical effects associated with control efforts could also be observed in both scenarios. Again, the details of the changes in burden following implementation of control will depend on both the core parameters described above, but also quantification of the impact of control efforts. If vaccine development is successful, deployment is likely to be targeted primarily at vulnerable groups (women of childbearing age) potentially followed by men (given evidence for sexual transmission). Such targeting is much less likely to result in the 'paradoxical effect' observed for rubella introduced into routine childhood immunization programs 5. However, over the longer term, some form of routine deployment of a ZIKV vaccine is likely to be considered should such a vaccine be developed (perhaps around age 9 years, as for HPV vaccine) and considerations of age incidence effects as for rubella re-emerge.

To conclude, the existing body of research into rubella provides a powerful starting point for thinking about the implications of teratogenic infections like ZIKV, but there are also many clear directions for further work. Overall, the invasion dynamics of teratogenic infections remain under-studied. More specifically, for ZIKV, theory building around existing frameworks for rubella could be deployed to identify core biological features required to characterize both the burden and how it will respond to control efforts, in both the long and short term 21. Sensitivity to core assumptions under varied demographic, epidemiological and control settings will delineate the critical aspects of the biology that must be known to best manage the burden of this infection. The magnitude and impacts of sterilizing immunity, as well as antigenic maps measuring cross-protection with other flaviviruses, and the magnitude of transmission, or R0 7, as well as likely impact of control efforts on transmission are likely to be key variables for robust inference into the outcome of management efforts for ZIKV.

## Competing Interest Statement

The authors have declared that no competing interests exist.

## Data Availability Statement

There is no raw data associated with this paper. The citation 7 was used to construct the models.
